# Nursing Students’ Experiences of Gratitude Journaling during the COVID-19 Pandemic

**DOI:** 10.3390/healthcare9111473

**Published:** 2021-10-30

**Authors:** Heesung Ko, Seryeong Kim, Eunjeong Kim

**Affiliations:** 1 Department of Nursing, Jesus University, Jeonju-si 54989, Korea; hsko@jesus.ac.kr; 2 Department of Education, Jeonju University, Jeonju-si 55069, Korea; jjy33@jesus.ac.kr; 3 Center for Teaching & Learning, Jesus University, Jeonju-si 54989, Korea

**Keywords:** gratitude journaling, nursing students, qualitative research

## Abstract

Gratitude journaling has been used to improve grateful disposition. However, there is only limited data available on its application experience. This study aimed to: (1) explore the experiences of nursing students who have participated in gratitude journaling; and (2) assess students’ views of gratitude journaling as a nursing intervention. This study implemented an eight-week program of gratitude journaling with fourth-year nursing students who took a mental health psychiatric nursing course at a South Korean university. Following the eight weeks, students reflected on their gratitude journaling experience in a reflective essay. Using content analysis, 53 essays were analyzed. Five categories were identified from the reflective essay, as follows: “A new beginning”, “The engine that motivates continued participation: gratitude sharing”, “The process driving change”, “Changes brought about by gratitude”, and “Self-reflection”. Based on this experience, nursing students believed that it is important to promote steady participation when administering gratitude journaling as a nursing intervention. The study findings suggest that the gratitude journaling not only helped with nursing students’ perspective, emotional, and behavioral aspects and stress management, but also provided an opportunity to advance a step further based on self-reflection.

## 1. Introduction

Gratitude, based on positive psychology, is conceptualized as a specific state of mind as well as a tendency. It is the perception of and accepting benefits from other people, objects, or nature as a gift and reacting to it with appreciation and joy [1]. A grateful disposition is defined as “a generalized tendency to recognize and respond with grateful emotion to the roles of other people’s benevolence in the positive experiences and outcomes that one obtains” [2]. People with a highly grateful disposition adopt positive views about themselves even during stressful or challenging situations, thereby promoting happiness and well-being [3].

While findings on the effects of gratitude on general health remain inconsistent, some studies have reported that gratitude has a positive impact on some aspects of physical health, such as cardiovascular health, inflammatory reactions, and sleep quality [4,5,6]. Gratitude is also associated with social, emotional, and psychological well-being, where it promotes social well-being by enabling individuals to form new relationships and maintain existing relationships [3]. People with a higher grateful disposition perceive that their positive experiences are attributable to other people’s help and react with gratitude and other positive pro-social emotions, contributing to high interpersonal competency [7]. Gratitude also promotes individuals’ perceived happiness and life satisfaction [3]. People with a highly grateful disposition have a well-developed schema for discovering the positive aspects and things they appreciate in various settings in daily life. These individuals find the silver lining in negative situations and react to these situations with positivity [8]. Further, gratitude was found to increase stress resistance [9]. Most existing Korean studies on gratitude have investigated the associations among gratitude, personality psychology, adaptation, subjective well-being, life satisfaction, and interpersonal relationships. Many other studies have also implemented and evaluated the effects of gratitude interventions on adults and elementary school students [10]. It has been reported that people with a highly grateful disposition experience fewer psychological problems such as depression, anxiety, and stress [11]. A grateful disposition has also been reported to serve as a protective factor against stress and depression and to mediate the relationship between these two factors [12].

Gratitude journaling, which refers to writing about objects, people, and events to boost one’s grateful disposition, is frequently utilized in gratitude-promoting programs [13,14,15]. During gratitude journaling, people periodically express and record their gratitude towards objects, people, or events. Emmons (2007) reported that while it is not easy to find things to be grateful for in the early days of gratitude journaling, people become more appreciative once they acknowledge the positive elements that exist in their life. This is because when one’s views are broadened and deepened through gratitude exercises, one can begin to see past adversities and focus on the “blessings” that are before them. This leads to an appreciation for things previously overlooked, which, in turn, leads to positive emotions. Thus, a newly acquired ability to feel grateful can encourage a virtuous cycle of behavior [16].

College students are in a transitional stage from adolescence to adulthood, and they face an array of stressful situations, such as adjusting to college life, maintaining good grades, managing interpersonal relationships, and gaining independence from their parents [17]. In particular, nursing students in Korea experience a high level of stress as they undergo an intensive curriculum, including clinical practicum, which prepares them for the national licensure examination to become nursing professionals [18]. Moreover, the increased proportion of online classes amid the COVID-19 pandemic that began in December 2019 has required students to adjust to a new learning environment that has escalated their academic stress even more [19]. Given the evidence that COVID-19 has an impact on the mental health of health care providers, nursing students can experience intensified stress owing to potential exposure to COVID-19 during clinical practicum, even if they do not provide direct patient care [20]. In this context, gratitude journaling may help in reducing stress and promoting positive emotions among nursing students.

Learning using a reflection journal provides an opportunity to connect and restructure various thoughts and feelings in acquiring and applying new pieces of knowledge [21]. Since reflection allows individuals to develop their views about a phenomenon, it makes the experience meaningful [22]. College is a period during which students are encouraged to engage in deep thinking and reflection, and therefore reflective writing can be educationally effective during this period [23]. From this perspective, reflective writing about their gratitude journaling offers nursing students an opportunity to comprehensively review their experiences and this writing data may uncover their vivid lived experiences of gratitude journaling. While gratitude journaling is known to effectively promote gratitude disposition, research identifying the specific paths and methods involved in this is still lacking [10]. It is also necessary to identify the factors that motivate continued participation in gratitude journaling. Hence, analyzing reflective essays can help to gain an understanding of the phenomenon. In this context, this study aimed to explore nursing students’ experiences of gratitude journaling and to investigate the usefulness of gratitude journaling during situations with high uncertainty, such as the COVID-19 pandemic, by analyzing reflective essays about their gratitude journaling experience.

## 2. Materials and Methods

### 2.1. Research Design

This qualitative study explored nursing students’ experiences with gratitude journaling by analyzing reflective essays about these experiences, using content analysis of responses to semi-structured essay questions distributed to the students.

### 2.2. Participants

Fourth-year nursing students from a university in Korea were enrolled in this study. Students who took a mental health psychiatric nursing course in the first semester of 2020 and kept a gratitude journal for eight weeks met the inclusion criteria.

### 2.3. Procedure

Students who took a mental health psychiatric nursing course in the first semester of 2020 were given an assignment to write a daily gratitude journal. Students were instructed to identify five things they were grateful for, five days a week for eight weeks. A total of 40 gratitude journal entries were written. The course was conducted online throughout the entire semester. On the first day of the course, the students were given a 30-min online orientation about the purpose and method of gratitude journaling. Students were given the option to keep a handwritten journal or use a mobile app for journaling. It was explained to the students that they could write the journal whenever they find something to be grateful for, or they could choose a time of day to write the journal. In addition, the students were instructed to form voluntarily their own groups within 5 people on social media to remind one another to journal. In the second week, the students watched a 30-min online video about the physical and mental effects of gratitude (about physical and emotional changes induced by gratitude and changes experienced when actually writing in a gratitude journal). To monitor their progress, students were instructed to take a photo or screenshot of their gratitude journal once every two to four weeks and upload it to the Learning Management System. The students were informed that the details of the journal need not be shown and that only the date of the journal and their five things should be shown. After eight weeks of gratitude journaling, the students were asked to write a reflective essay about their gratitude journaling experience. A semi-structured list of questions was provided prior to the beginning of gratitude journaling. The following questions were asked:

- Describe your overall thoughts and feelings about gratitude journaling.
- For which areas (e.g., thoughts, emotions, stress management) did you find gratitude journaling helpful?
- What are some things you realized while keeping a gratitude journal?
- What factors should a nurse consider when administering a gratitude journaling intervention?

A total of 128 students, divided into two classes, took the course. All of the students submitted their reflective essays about gratitude journaling. Of 54 essays from the students who consented to the use of their data, one incomplete essay was excluded, and 53 reflective essays were analyzed.

### 2.4. Ethical Considerations

This study was approved by the Korean Public Institutional Review Board, which is designated by Ministry of Health and Welfare (No. P01-202101-23-003). To confirm the participants’ voluntary consent to the use of their previously submitted data for research, a written consent was obtained in the second semester of 2020, after the grades for the previous semester were finalized. First, information about the study was posted on an online community for fourth-year students. The students were instructed to complete the written consent form and submit it to the researcher in person or via email or text message should they wish to participate in the study. All participants were given a mobile drink voucher.

### 2.5. Analysis

In this study, the semi-structured reflective essays about the students’ journaling experiences over eight weeks were analyzed via content analysis. The responses to three questions (overall thoughts and feelings about gratitude journaling, how gratitude journaling has helped with thoughts, emotions, and stress management, and newly realized things through gratitude journaling) were partially redundant, and therefore, these responses were analyzed as “gratitude journaling experience”. Content analysis is a systematic and objective technique for describing qualitative content, systematically categorizing information, and investigating the contents of the recorded information [24].

Three researchers read the written statements repeatedly to review whether the statements were relevant to gratitude journaling. Next, the words, phrases, and sentences were separated and coded into units of content analysis. Then, similar content was clustered and redescribed as an in vivo coding approach while ensuring that the original meanings were retained to select significant statements. In the third step, similar significant statements were clustered and conceptualized into an overarching code. Each theme cluster was named, and the contents of the themes and categories were reviewed. Finally, the incidences and frequencies of statements about things nurses should consider when administering long-term gratitude journaling interventions were identified.

To establish the rigor of the study, we considered credibility, fittingness, auditability, and confirmability as proposed by Sandelowski (1986) [25]. Gratitude journal data from all consenting students were analyzed with the exception of one incomplete journal, and credibility was ensured through continuous discussions among the researchers at each stage of the analysis. Fittingness was ensured by confirming the study results with two participants. Auditability was ensured by describing the data analysis process and citing the direct quotes from the raw data in the text. Finally, confirmability was ensured by minimizing researchers’ bias and attempting to generate results that reflect participants’ experiences.

## 3. Results

Of the 53 participants, 51 were female (96.2%) and two were male (3.8%). All of them were fourth-year nursing students, and the mean age was 22.9 ± 5.03 years. The sizes of the small social media groups ranged from two to five people, with an average of 3.6 students per group. There were more students who wrote the journals by hand (*n* = 33, 62.3%) than those who used the mobile app (*n* = 20, 37.7%) (Table 1).

### 3.1. The Gratitude Journaling Experience

Through an analysis of the reflective essays about gratitude journaling among the nursing students, 156 meaningful statements in 14 sub-themes under five themes emerged. The five themes were: (1) a new beginning, (2) the engine that motivates continued participation: gratitude sharing, (3) the process driving change, (4) changes brought about by gratitude, and (5) self-reflection (Table 2).

#### 3.1.1. A New Beginning

- “Writing about gratitude is awkward and difficult”

Initially, the participants had difficulty in immediately coming up with things they were grateful for. They described completing this assignment methodically and without being fundamentally motivated. Hence, the students primarily wrote about things they had received from other people and they often skipped a day of journaling if they felt like there was nothing to be grateful for. This process was generally perfunctory.

*At first, I wasn’t sure about how and what I should write about in my gratitude journal. So, I simply wrote about the help I received from someone and material things that I was grateful for. When writing in this manner, sometimes I had a lot to write about, and I also skipped a day when I did not have anything to write about.*

(Participant 3)

*At first, I didn’t know what to do. I was not really grateful for anything, and I could only come up with just one or two things if I thought hard. So there were days when I wrote 1–3 days’ worth in a single day.*

(Participant 4)

- “Having hopeful expectation about the changes to come”

In contrast to some participants who felt awkward when writing about things they were grateful for, some participants engaged in gratitude journaling while seeking a means of stress management. Despite this being an assignment for a course, they kept the journal earnestly from the beginning, considering it as an activity that would benefit themselves with hopeful expectations for positive changes they hope to experience during the process.

*I was finding my own strategies as I contemplated how I should improve my self-esteem and cope with situations that I will face in the future. During that time, I was given this gratitude journaling assignment by my professor, and instead of considering it as an assignment, I decided to keep the journal for my own good.*

(Participant 23)

*When I first found out that gratitude journaling is an assignment for the entire semester, I did not really feel overwhelmed, but I was actually hopeful for the changes that I will see in my life through gratitude journaling.*

(Participant 33)

- “Gradually becoming familiar with the process”

The participants described how, at first, it was difficult to find things to be grateful for, but over time, thinking about these things throughout the day became more routine, and they began to notice more things to be grateful for. The participants were able to maintain the journal regularly.

*After exactly one week, things that I was grateful for began to pop up in my head. Waking up in the morning, washing my face and hair, eating food… I realized that I do not really have to think deeply. And so this became more fun, I had more to write about, and on some days, I had too much to write, and there was not enough (space).*

(Participant 4)

*However, by about one week or two weeks later, I automatically began to think that ‘Oh, I am grateful for this today’. I can be grateful for this kind of thing, too, when something happened.*

(Participant 6)

#### 3.1.2. The Engine That Motivates Continued Participation: Gratitude Sharing

- “Learning from others’ gratitude”

Watching a video of the effects of gratitude motivated the participants with gratitude journaling. Further, sharing their gratitude journals with their group members or sharing their gratitude with each other fostered close social bonds as they learned about each other’s values and thoughts. Moreover, participants were able to view things from a different perspective through others’ gratitude stories, and even though they could not meet in person due to the pandemic, the small groups served as a channel to encourage and support one another.

*I watched a video about gratitude journaling, and this served as an opportunity for me to start writing my gratitude journal with a new mindset. Instead of simply being grateful for others, I began to be grateful for the things I have.*

(Participant 3)

*During gratitude journaling, I met with two friends and we shared about grateful things, and I even felt grateful for being able to listen to what others are grateful for. So, I was so thankful for this time and was so happy. I think this gratitude is something I would have never felt if I had not written a gratitude journal.*

(Participant 15)

- “Being able to continue journaling thanks to colleagues”

Participants claimed that keeping a gratitude journal for eight weeks was not easy but forming small groups for sharing and encouragement not only motivated them to continue with gratitude journaling, but also to enjoy the process of journaling.

*The reason I was able to enjoy this gratitude journaling was that we did it together. By sharing important things about each other’s lives, we discovered new things about each other, and our gratitude doubled through positive feedback.*

(Participant 53)

*I think my group members played a huge role in my ability to continue keeping a gratitude journal. They endlessly encouraged me, wishing me to have only grateful things in the future.*

(Participant 24)

#### 3.1.3. The Process Driving Change

- “Thoughts are becoming more flexible and broadened”

There were a few steps that led to various small changes in the students’ thinking, including thoughts, emotions, and behaviors, by identifying the things they were grateful for and recording them every day. First, as the participants focused on gratitude, they became more engaged in positive and flexible thinking and became more lenient toward others as they put themselves in others’ shoes. Further, they found that their thoughts were broadened in areas that they had not previously perceived.

*Even in the same situation, I have become able to think about ‘Maybe?’ for problems that I first asked ‘Can it?’ before writing the gratitude journal. I feel like I have found my lost positivity.*

(Participant 16)

*My thoughts have become more flexible. When I make a mistake or when someone does something rude, I now think ‘Well, things happen’.*

(Participant17)

*As I began to make being grateful for the little things in life a habit, I was able to strengthen my ability to view the world with a broader perspective.*

(Participant 3)

- “Observing daily life more closely”

As the participants consciously searched for things to be grateful for in their ordinary lives, they became more observant of things that they had neglected or not noticed before. This led to further gratitude in a virtuous cycle.

*When I look at objects, I became more observant of things that I had neglected before and began to find gratitude from even little things.*

(Participant 29)

*Because I kept thinking about gratitude (throughout my day) to write the gratitude journal, I observed even little things more closely and was able to find grateful things from various perspectives. Especially, I felt good when I found grateful things in my daily life, and this helped me maintain a good mood and work harder even in negative situations.*

(Participant 49)

- “Intentionally striving to remember to be grateful”

Students began to intentionally capture and remember meaningful and pleasant moments. By doing so, they were able to focus on the present and learn the importance and value of the process, as opposed to the outcome of a task.

*Recently, I received a small compliment from a friend, and usually I’d just let it go right after but while writing the gratitude journal, I left a record of that compliment in my journal and was able to think about the compliment for longer; I came to think that I received good influence from that friend.*

(Participant 15)

*Writing a gratitude journal seems to help me recall happy memories as I recollect things in life that I took for granted and so it helps me live a positive life.*

(Participant 32)

#### 3.1.4. Changes Brought about by Gratitude

- “A change of perspective”

Gratitude journaling enabled participants to appreciate the little and ordinary things in life and transformed their negativity into positivity. Most of all, the participants were now able to be grateful for their own existence as well as others.

*I used to always have a pessimistic view of my situations, but while writing a gratitude journal, I realized that my life is a pretty good life and that situations that I thought could not be worse were actually thankful situations.*

(Participant 44)

*I learned to appreciate myself. At first, I tried to find things to be grateful to other people or their surroundings. However, I thought that being grateful for myself, who is the center of all this, is very important too. The more I appreciated myself, I tried to become that person commensurate with the appreciation, and although not all things I do can be recognized by others, I realized that I am the one who would understand my hard work the most. As I thanked myself, my self-esteem increased, and I was able to learn how to encourage myself.*

(Participant 30)

- “Emotional changes”

A transformation of perspective led to positive emotions. The participants were able to identify their emotions as they wrote in the gratitude journal and expressed or regulated their emotions even when they had negative feelings.

*I developed the ability to quickly change my thoughts when I have negative emotions. Even when I faced hardship, I was able to route my mind that had been set on negative thoughts to other thoughts by thinking about grateful things in the past. And, I acquired the strength to get back on my feet again with hopeful expectations for other grateful things to come.*

(Participant 49)

*Writing a gratitude journal helped me wrap up the day and feel organized. While writing the journal, I would read over things I wrote in the past and recollect that Oh, I was really grateful for this back then… And it made me feel grateful for my current surroundings again.*

(Participant 26)

- “Behavioral changes”

Through gratitude journaling, the participants adopted a more positive attitude towards other people and expressed their gratitude. They aimed to show more generosity towards people and wanted to embody someone who would become an object of gratitude for another.

*Expressing gratitude was not as difficult as I thought it would be, and it brought about many changes. At first, I just wrote in the journal and thought about it alone, but as I continued writing, I wanted to express this gratitude. After I expressed my gratitude, I felt like it had a lot of positive influence on people around me as well as myself.*

(Participant 39)

*As I tried to express my gratitude even in situations that would not normally require me to say thank you, I’ve seen many people around me feel happy. The other person that I thanked would try to help me more, and I would also try to reciprocate that kindness, and naturally, I came to think a lot about living life with others.*

(Participant 26)

#### 3.1.5. Self-Reflection

- “Contemplating myself”

Remembering and recording things the students were grateful for served as an opportunity to reflect on themselves. As they were able to more clearly understand their thoughts and emotions, they thought more deeply about themselves, and this further enriched their lives.

*As I read over my previous gratitude journals, I felt that I strived to take responsibility for my roles even during difficult times due to COVID-19 and worked hard to solve my problems. I used to always blame myself for everything, but as I read over my gratitude journal, I learned to give myself recognition a little bit.*

(Participant 14)

*While writing a gratitude journal, I felt the importance of ‘giving’. The gratitude journal enriched my life and gave me a chance to introspect.*

(Participant 19)

- “Daily reflections becoming a routine”

Most participants wrote in their gratitude journal as they wrapped up the day, and by engaging in introspection with a focus on things they were grateful for, they were able to gain strength by imagining themselves as better people, and gratitude journaling was established as a positive routine.

*When I wrote my gratitude journal at the end of the day, I re-faced the emotions I had that day and introspected. As I thought about my gratitude for people and thought about myself, I was able to imagine myself tomorrow.*

(Participant 17)

*Personally, I wrote my gratitude journals at the same time of the day in the evening, and I think writing the journal at the same time of the day as a ritual for opening up a day or ending a day is more effective. In particular, writing the journal in the evening was really good for me because I was able to relax time as I reviewed my day and recollected grateful things and pleasant thoughts in detail.*

(Participant 39)

- “Continuing to engage in valuable experiences together”

The valuable experience of self-reflection through gratitude journaling motivated the participants to continue writing in the journal or to recommend gratitude journaling to other people.

*At first, I simply thought of it as an assignment, but now it has become a healing program for me so much that I introduced it to a friend suffering from depression. So, I am really grateful for learning about gratitude journaling.*

(Participant 44)

*As I am excessively critical, I will continue writing a gratitude journal with a hopeful expectation that making this a daily routine would help me discover other facets of life.*

(Participant 31)

### 3.2. Factors to Be Considered by Nurses When Administering Gratitude Journaling as a Nursing Intervention

The most common response regarding the factors to be considered when implementing gratitude journaling as a nursing intervention based on the eight-week experience was to encourage steady participation through support, encouragement, motivation, feedback, and gratitude sharing (69.8%), followed by preparing the participants to help them accept gratitude journaling (35.9%), preparing nurses themselves as therapists (34.0%), informing participants of the purpose and method (34.0%), and fostering a physical and temporal environment (15.1%) (Table 3).

## 4. Discussion

The experiences of the fourth-year nursing students’ eight weeks of gratitude journaling were summarized into five themes: (1) a new beginning, (2) the engine that motivates continued participation: gratitude sharing, (3) the process driving change, (4) changes brought about by gratitude, and (5) self-reflection. The factors worth considering when administering gratitude journaling as a therapeutic nursing intervention were also summarized.

In regard to the first theme “a new beginning”, the participants were new to gratitude journaling and felt burdened and obligated to write in the journal regularly at first. Although the participants were given an orientation about how to write in the gratitude journal, they were unfamiliar with recollecting and recording things they were grateful for and thus had difficulty in the beginning. As reported by a previous study, it is difficult to keep a gratitude journal at first, as perceiving certain things in one’s life as blessings requires a new way of thinking. Individuals feel most comfortable expressing gratitude once they realize that their lives contain small things and moments worth being grateful for, which takes some learning. In other words, participants need some time to adapt to this otherwise unfamiliar activity [16]. Participants who began gratitude journaling with hopeful expectations considered it an opportunity for self-change, imagining the positive changes they will see in the future. Both cases gradually became familiar with gratitude journaling after some time. Thus, when planning interventions utilizing gratitude journaling, various motivation strategies should be employed to reduce the participants’ feelings that writing is a burden. For example, instead of beginning to write about what they are grateful for, they can be encouraged to first express their gratitude verbally or be given the opportunity to listen to others’ gratitude.

The second theme was “the engine that motivates continued participation: gratitude sharing”. The participants formed small groups on social media to share their personal experiences during the COVID-19 pandemic, to encourage one another to journal regularly, and share their stories of gratitude with each other. This motivated their continued participation in gratitude journaling. During this process, they were inspired by others’ gratitude stories and learned how to practice gratitude further. A group-based gratitude-promoting program provides an opportunity for members to practice expressing gratitude, observe how others do it, and to hold each other accountable for continuous participation [26]. Even though the participants did not meet in person, sharing their gratitude stories through social media helped them partially reap the benefits of group therapy. Subsequent studies may consider comparing the effects of individual gratitude journaling and group gratitude journaling based on this sharing. This is an implication for future studies.

The third theme was “the process driving change”, where some steps led to positive changes in thoughts, emotions, and behaviors through eight weeks of gratitude journaling. The participants took a new approach to finding things to be grateful for in their daily lives and broadened their thoughts and increased the flexibility of their thinking. Further, they came to observe things in their daily lives more closely that they had neglected or not noticed before, and they intentionally tried to remember pleasant things. This is similar to the process of discovering unique outcome techniques in narrative therapy. As narrative therapy produces therapeutic effects by having participants seek “exceptions” in a problem and reinforce the process, it seems that the participants experienced self-treatment through a process of searching for grateful things and intentionally trying to remember them [27]. In addition, this is similar to previous reports that people with a highly grateful disposition have well-developed schemas to find positive aspects in various environments, identify objects of gratitude, find positive features even in negatively perceived situations, and reinterpret them in their favor [8,28].

The fourth theme was “changes brought about by gratitude”. The participants experienced actual changes in their perspectives, emotions, and behaviors through gratitude journaling. Gratitude is strongly correlated with positive emotions [3,29], promotes happiness and health, and contributes to reducing negative emotions and problems [3]. The participants not only experienced positive emotions through gratitude journaling, but also developed the ability to recognize and control their emotions, which is consistent with previous findings that state how focusing on things that bring gratitude for at least a few minutes a day can reduce symptoms contributing to anxiety and depression [26]. Furthermore, gratitude manifests as a pleasant reaction to external factors after recognizing the benefits received from them [30]. The participants of this study also showed behavioral changes, such as expressing gratitude for their own existence and for others and a motivation to give back to others, as they felt grateful for what they had received. The fact that writing about gratitude promoted prosocial behavior by increasing positive emotions and empathetic thinking during the COVID-19 pandemic [31] is in line with our results.

The final theme was “self-reflection”. Most of the participants wrote in their gratitude journal at the end of the day, as opposed to recording immediately after experiencing gratitude. Participants then intentionally recollected moments of gratitude throughout the day and spent time introspecting. Thus, gratitude journaling was established as a day’s wrap-up routine, and consistently keeping the journal served as an engine for habitually continuing the gratitude journal. Journaling is an effective tool for introspection among college students [32]. This is because individuals develop a unique schema; that is, as they focus on things for which they are grateful for the purpose of journaling, they undergo a process of reinterpretation whereby they try to find things to be grateful for even in negative situations. It seems that self-treatment occurs when one engages in deeper reflection about oneself and their day, and imaging their next day becomes routine. After realizing how gratitude journaling shifted their thought processes, emotions, behaviors, and relationships with others through eight weeks of practice, the participants wanted to continue the practice and recommended gratitude journaling to their friends and family. They developed expectations for positive changes they aimed to experience through the long-term practice of gratitude journaling. Emmons (2002) stated that gratitude is a private but also communal experience, and thus can go beyond social convention [33], which is similar to our findings where individuals who experienced gratitude spread the practice to people around them.

Nursing students stated that encouraging consistent participation is important when utilizing gratitude journaling as a nursing intervention. This is speculated to be because the students themselves experienced positive attitudes toward life after eight weeks of participation in gratitude journaling. A previous study showed that a two-week gratitude journaling intervention led to improved psychological and emotional well-being [34], and our results suggest that a longer intervention was positively rated by the participants. Furthermore, factors such as nurses’ readiness as therapists, patient readiness, and the instructions about the purpose and method of gratitude journaling should be taken into consideration when administering a gratitude journaling intervention.

One strength of this study is that it confirmed that gratitude journaling can help nursing students to manage their thoughts, emotions, and stress as well as promoting self-contemplation. Furthermore, the study results highlight the importance of sharing groups and consistent participation in continuing to engage in gratitude journaling.

This study is limited in that it analyzed gratitude journaling experiences and relevant essays of nursing students in a single university in Korea. A more diverse study population and in-depth interviews with volunteers would be needed to generalize the study findings. In addition, a mixed methods design could also be considered to gain a comprehensive understanding of the effects of gratitude journaling.

## 5. Conclusions

Gratitude journaling not only appeared to help nursing students with perspective, emotional, and behavioral aspects of their lives while navigating the challenges of their education and the COVID-19 crisis, but also appeared to assist with stress management. This was accomplished through the promotion of abstract thinking or a new approach to noticing and acknowledging things in one’s life to be grateful for. The exercise also encouraged participants to focus on positive aspects in various situations and provided an opportunity for nursing students to advance a step further through introspection and self-reflection. The experiences of the participants in this study suggest that gratitude journaling interventions, when designed with effective factors in place that facilitate and encourage continuous practice, may serve as an effective means for assisting nursing students with stress management.

## Figures and Tables

**Table 1:** General Characteristics of Participants (*n* = 53).

Characteristic	*n* (%) or M ± SD
Gender	
Male	2 (3.8%)
Female	51 (96.2%)
Age	22.9 ± 5.03
Number of sharing group members	3.6 ± 1.12
Hand-written journal	33 (62.3%)
Mobile app	20 (37.7%)

**Table 2:** Themes that emerged from the gratitude journaling experience.

Themes	Sub-Themes
A new beginning	Writing about gratitude is awkward and difficult
	Having hopeful expectations about the changes to come
	Gradually becoming familiar with the process
The engine that motivates continued participation: gratitude sharing	Learning from others’ gratitude
	Being able to continue keeping the journal thanks to colleagues
The process driving change	Thoughts becoming more flexible and broadened
	Observing daily life more closely
	Intentionally striving to remember to be grateful
Changes brought about by gratitude	A change of perspective
	Emotional changes
	Behavioral changes
Self-reflection	Contemplating myself
	Daily reflection becoming a routine
	Continue engaging in valuable experiences together

**Table 3:** Factors to be considered by therapists regarding a gratitude journaling intervention (*n* = 53).

Categories	*n* (%)
Prepare oneself as the therapist	18 (34.0%)
Foster physical and temporal environment	8 (15.1%)
Inform participants of the purpose and method	18 (34.0%)
Encourage steady participation	37 (69.8%)
Confirm participants’ readiness	19 (35.9%)

## Data Availability

The data presented in this study are available upon reasonable request from the authors. The data are not publicly available due to privacy or ethical restrictions.
